# RETRACTION: Hyperspectral Imaging‐Based Erythema Classification in Atopic Dermatitis

**DOI:** 10.1111/srt.70282

**Published:** 2025-10-21

**RETRACTION**: S. Kye, and O. Lee, “Hyperspectral Imaging‐Based Erythema Classification in Atopic Dermatitis,” *Skin Research and Technology* 30, no. 3 (2024): e13631, https://doi.org/10.1111/srt.13631.

The above article, published online on 23 February 2024 in Wiley Online Library (wileyonlinelibrary.com), has been retracted by agreement between *Skin Research and Technology*; and John Wiley & Sons Ltd. The article was accepted for publication following an insufficient peer review process that does not meet the standards of the journal's peer review policy. Following post‐publication review, the article was deemed unsuitable for publication due to the absence of critical methodological details necessary for interpreting and replicating the findings, and because the conclusions are not sufficiently supported by the data presented. Accordingly, the article has been retracted. The authors have been informed of the retraction decision but were unavailable for final confirmation.

